# Assessment of endometrial receptor systems for PPPM approach for endometrial hyperplasia in reproductive age women

**DOI:** 10.1186/1878-5085-5-S1-A39

**Published:** 2014-02-11

**Authors:** Vadym M Goncharenko, Vasyl A Beniuk, Yaroslav M Vyniarskyi, Sergiy M Bashynskyi, Rostyslav V Bubnov

**Affiliations:** 1 Clinical hospital "Pheophania", Kyiv, Ukraine; 2 Bogomolets National Medical University, Third Department of Obstetrics and Gynecology, Kyiv, Ukraine; 3 Institute of Pathology, Helios Klinik, Berlin, Germany

## Scientific objectives

Endometrial hyperplasia (EH) is significant in gynecological morbidity of reproductive age women, has high risk of malignancy and associated with menstrual irregularities, dysfunctional uterine bleeding and anemia. The incidence of endometrium adenocarcinoma ranks first among genital malignancies, in recent years has tended to increase in many countries, including Ukraine. EH has tended to increase in many countries, including Ukraine, in recent years. The response to hormone therapy and prognosis of endometrial hyperplastic processes in women is largely determined by receptor status, which depends on the clinical stage and degree of histological differentiation of EH.

## The aim

was to determine the state of endometrial receptor systems in each case of endometrial hyperplasia using immunohistochemical methods to perform personalized treatment strategy.

## Materials and methods

We included to the study 119 women aged 25 to 45 years (the average was 38, 0 ± 2, 3 years) treated in Hospital "Pheophania", assessed to: group 1 (n = 41) with endometrium glandular-cystic hyperplasia; group 2 -patients with endometrial polyps (n = 53); and group 3 (n = 21) - with atypical EH; the control group included 22 women, who underwent in vitro fertilization due to tubal origin infertility. All patients underwent extensive general clinical examination, which included clinical and biochemical blood tests, blood tests for HIV, RW, HBS-Ag and HCV-Ag, clinical urine tests, ECG, ultrasound, chest X-rays, a study of vaginal biotope (microflora). The EH was diagnosed using transvaginal ultrasound followed by hysteroresection. The resulting material was subjected to histological study to determine the receptor of the endometrium cells applying immunohistochemical method.

## Results

In patients with glandular hyperplasia we registered the sharp increase of estrogen receptors in the endometrium (75.6% in epithelial cells and 30.9% in stroma, in control group 43.3% and 29.6%, respectively). Analysis of the distribution of receptors for progesterone showed them a slight increase in the endometrium and in the stroma (1.3 times). In endometrial polyps group we found significant increase of estradiol receptors in stroma (48.2% vs 29.6% in controls). In patients with atypical endometrial hyperplasia the significant increase in the content of progesterone receptors in endometrial stroma - 81.8%. The proliferative marker Ki-67 was determined significant increased in 40-50% in the epithelium of the glands in the absence of changes in the stroma compared with glandular hyperplasia.

## Outlook and expert recommendations

Thus, our analysis of the ratio of receptor in tissue and endometrial stroma in patients of observation allows to conclude as follows:

• These receptor combination patterns are **predictive criteria** for determining the subsequent treatment strategy as the method of screening for uterine cancer pathology.

• Each individual pathological pattern of the definition of receptors and their relationship further defines **personalized** pathogenic tactics.

• The sharp variations in the receptor status of the endometrium can be interpreted as a risk factor for development of genetic mutations and carcinogenesis.

• It is recommended to create a project to perform a good evidence study of determining relationships in endometrial receptor system to complement the diagnostic algorithm that will allow to conduct pathogenetic therapy.

